# Turning Over a New Leaf: Cannabinoid and Endocannabinoid Modulation of Immune Function

**DOI:** 10.1007/s11481-015-9615-z

**Published:** 2015-06-09

**Authors:** Guy A. Cabral, Thomas J. Rogers, Aron H. Lichtman

**Affiliations:** Department of Microbiology and Immunology, Virginia Commonwealth University, Richmond, VA 23298 USA; Center for Inflammation, Translational and Clinical Lung Research, Center for Substance Abuse Research, Temple University School of Medicine, 3500 N. Broad Street, Philadelphia, PA 19140 USA; Department of Pharmacology and Toxicology, Virginia Commonwealth University, Richmond, VA 23298 USA

**Keywords:** Cannabinoids, Cannabinoid receptors, Endocannabinoids, Immune modulation, Neuroimmune effects, Marijuana, Phytocannabinoids

## Abstract

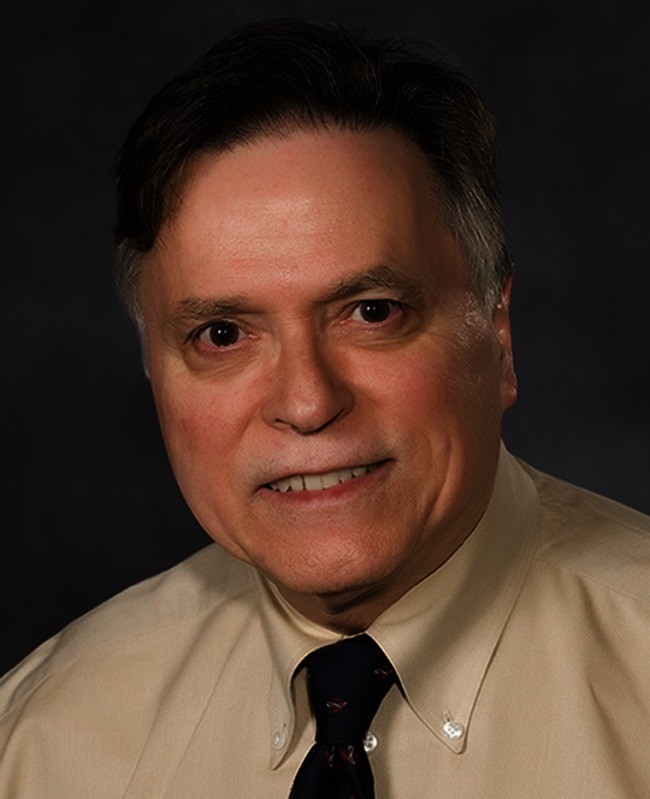

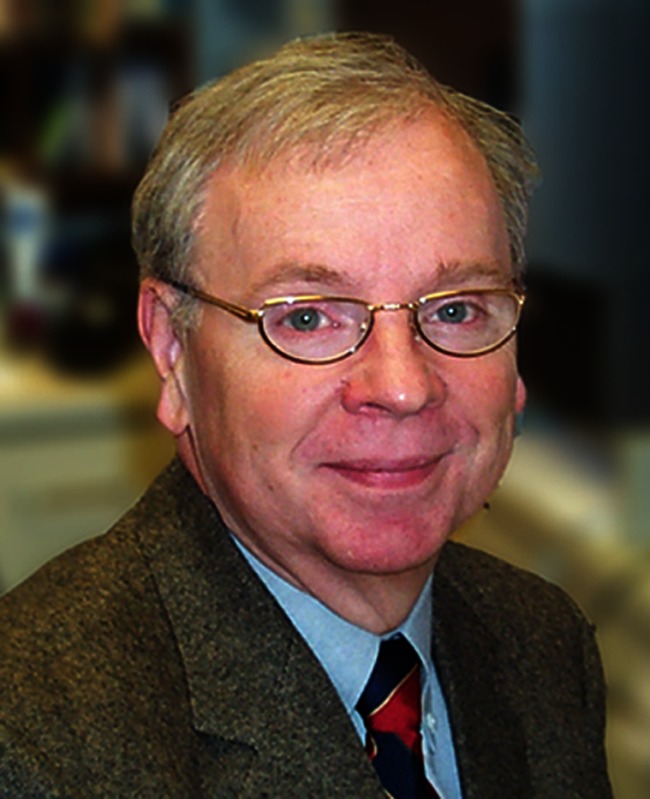

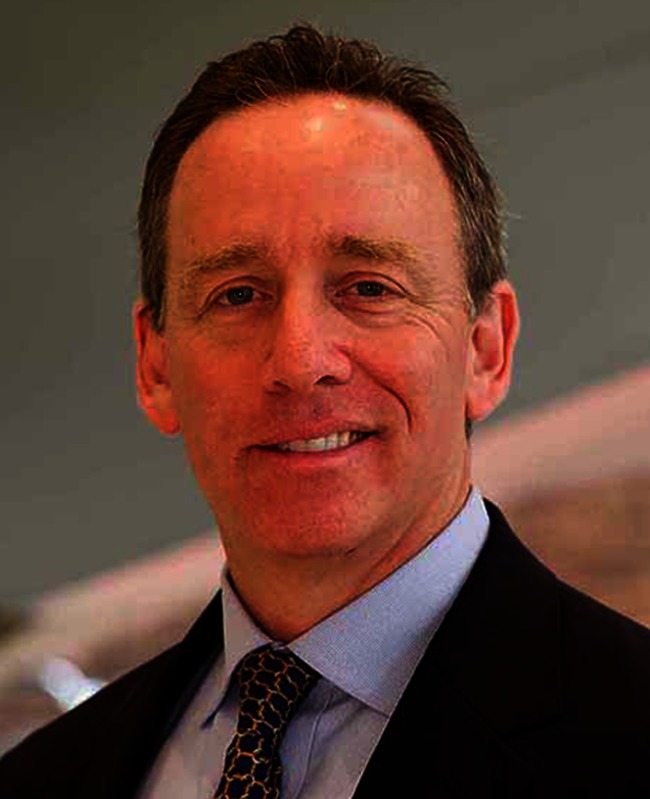

Cannabis is a complex substance that harbors terpenoid-like compounds referred to as phytocannabinoids. The major psychoactive phytocannabinoid found in cannabis ∆^9^-tetrahydrocannabinol (THC) produces the majority of its pharmacological effects through two cannabinoid receptors, termed CB_1_ and CB_2_. The discovery of these receptors as linked functionally to distinct biological effects of THC, and the subsequent development of synthetic cannabinoids, precipitated discovery of the endogenous cannabinoid (or endocannabinoid) system. This system consists of the endogenous lipid ligands *N*- arachidonoylethanolamine (anandamide; AEA) and 2-arachidonylglycerol (2-AG), their biosynthetic and degradative enzymes, and the CB_1_ and CB_2_ receptors that they activate. Endocannabinoids have been identified in immune cells such as monocytes, macrophages, basophils, lymphocytes, and dendritic cells and are believed to be enzymatically produced and released “on demand” in a similar fashion as the eicosanoids. It is now recognized that other phytocannabinoids such as cannabidiol (CBD) and cannabinol (CBN) can alter the functional activities of the immune system. This special edition of the Journal of Neuroimmune Pharmacology (JNIP) presents a collection of cutting edge original research and review articles on the medical implications of phytocannabinoids and the endocannabinoid system. The goal of this special edition is to provide an unbiased assessment of the state of research related to this topic from leading researchers in the field. The potential untoward effects as well as beneficial uses of marijuana, its phytocannabinoid composition, and synthesized cannabinoid analogs are discussed. In addition, the role of the endocannabinoid system and approaches to its manipulation to treat select human disease processes are addressed.

## Introduction

In this special edition of the Journal of Neuroimmune Pharmacology, we present a collection of cutting edge articles on the medical implications of marijuana use and the functionality of the endocannabinoid system. It is our goal to provide an unbiased assessment of the state of research related to this topic and we have solicited articles from leading researchers in the field. The seven reviews and ten original articles in this special themed edition of the Journal describe the role of phytocannabinoids and the endocannabinoid system on neuroinflammatory processes in in vitro, laboratory animal, and human systems.

Translating in vitro and in vivo results derived from experimental animals to the human condition is fraught with substantial challenges. Marijuana users may consume other drugs that affect immune function, complicating our understanding of the relative contribution of a distinct phytocannabinoid. Furthermore, because marijuana contains a plethora of phytocannabinoids and other classes of chemicals, attributing a specified action to a single constituent is difficult. This complexity is further augmented by emerging scientific data that show that distinct phytocannabinoids may activate immune cells by receptor-mediated as well as by non-receptor-mediated modes. In order to garner insight into the potential linkage between marijuana use in humans and compromised immune function, investigators have resorted to the use of purified synthetic phytocannabinoid preparations in cell culture models or in experimental animals.

## The Diversity of Cannabinoids

The discovery of the endogenous cannabinoid (endocannabinoid) system and concomitant explosion of basic knowledge pertaining to this system and cannabinoid pharmacology, combined with the controversy regarding potential medical benefits of cannabis versus its abuse and dependence liability, contribute to the impetus for disseminating the science appearing in this special edition. The long history of the use of cannabis for therapeutic and other purposes has been the subject of many reviews (Mechoulam et al. 1991; Russo 2007). Likewise, a detailed overview communicating the components of the endogenous cannabinoid system appears elsewhere (Blankman and Cravatt 2013; Howlett et al. 2011). To date, two cannabinoid-based medications have gained approval by the Food and Drug Administration, Marinol (dronabinol or ∆^9^-tetrahydrocannabinol (THC)), the primary psychoactive constituent in cannabis, and Cesamet (nabilone), a synthetic cannabinoid (Pertwee 2009; Rahn and Hohmann 2009). These two medications have been approved for the treatment of chemotherapy-induced nausea and emesis. Marinol also may be prescribed as an appetite stimulant to treat cachexia in AIDS patients. A third medication, Sativex, consists of a sublingual spray formation that contains equivalent concentrations of THC and cannabidiol (CBD) that are extracted from cannabis and has been approved in many countries to relieve spasticity in multiple sclerosis (MS) patients (Syed et al. 2014). In this special issue, we describe key substances present in cannabis, their biological properties, and mechanisms of action. We also review the interdisciplinary research leading to our understanding of cannabinoid- and endocannabinoid-mediated modulation of immune function within the nervous system.

## Cannabinoid Receptors

Cannabis is a complex substance that harbors terpenoid-like compounds collectively referred to as phytocannabinoids. The primary psychoactive constituent of cannabis, THC (Gaoni and Mechoulam 1964), produces the majority of its pharmacological effects through two cannabinoid receptors, termed CB_1_ (Devane et al. 1988; Matsuda et al. 1990; Herkenham et al. 1990) and CB_2_ (Munro et al. 1993). These two receptors share approximately 44 % amino acid homology (Munro et al. 1993). Both receptors have seven transmembrane domains, are coupled to G-inhibitory proteins, and are linked to signaling cascades that may involve adenylyl cyclase and cAMP, mitogen-activated protein (MAP) kinase, and the regulation of intracellular calcium (Howlett 2002). CB_1_ is expressed heterogeneously throughout the nervous system (Herkenham et al. 1991) as well as in other organ systems (Gerard et al. 1991). CB_1_ is predominantly responsible for the psychoactive effects of THC, and the stimulation of this receptor plays a role in regulating pain, stress responses, energy regulation and lipogenesis, and immune function. CB_2_ is primarily associated with immune function and is expressed on immune cells, including microglial cells within the nervous system, but its expression in the CNS is of a much smaller magnitude than that of CB_1_.

Cannabinoids bind to other receptors besides CB_1_ and CB_2_ (Breivogel et al. 2001; Di Marzo et al. 2000; Jarai et al. 1999), suggesting the existence of additional cannabinoid receptors or simply other binding sites (i.e., “off targets”). Included among these candidate cannabinoid receptors is GPR55, a seven-transmembrane G protein-coupled receptor first cloned and identified in silico from an expressed sequence tags database (Baker et al. 2006; Pertwee 2007; Sawzdargo et al. 1999). GPR55 is activated by THC and CBD, certain synthetic cannabinoids, and the endogenous cannabinoids N-arachidonoylethanolamine (anandamide; AEA) and 2-arachidonoylglycerol (2-AG) (Ryberg et al. 2007). However, unlike CB_1_ and CB_2_, GPR55 is coupled to a G-alpha (Gα) protein instead of a Gi/o protein (Ryberg et al. 2007), is not activated by the synthetic cannabinoid receptor agonist WIN 55212–2, and increases intracellular calcium levels upon activation (Lauckner et al. 2008). However, to date, a novel non-CB_1_, non-CB_2_ cannabinoid receptor that meets rigid pharmacological and functional criteria (i.e., is selective for cannabinoid ligands) has yet to be cloned and characterized at the molecular level (Breivogel et al. 2001; Di Marzo et al. 2000; Jarai et al. 1999; Wiley and Martin 2002; Pertwee et al. 2010).

## Endogenous Cannabinoids

The discovery of cannabinoid receptors that mediate the actions of THC and synthetic cannabinoids, precipitated the search for the endogenous ligands that bind these receptors. AEA (Devane et al. 1992) and 2-AG (Mechoulam et al. 1995; Sugiura et al. 1995) represent the primary endogenous ligands that bind and activate CB_1_ and CB_2_. Endocannabinoids have been identified in immune cells such as monocytes, macrophages, basophils, lymphocytes, and dendritic cells (Matias et al. 2002). These endocannabinoids are believed to be enzymatically produced and released “on demand” in a similar fashion as the eicosanoids. AEA and 2-AG are rapidly hydrolyzed by fatty acid amide hydrolase (FAAH; (Cravatt et al. 2001; Cravatt et al. 1996)) and monoacylglycerol lipase (MAGL (Dinh et al. 2002a; 2002b)), their respective chief degradative enzymes, though other enzymes play a role in endocannabinoid metabolism (Blankman et al. 2007; Hermanson et al. 2014). FAAH also hydrolyzes other bioactive fatty acid amides (Cravatt et al. 2001), such as N-palmitoylethanolamine and oleoylethanolamide each of which has been reported to possess anti-inflammatory actions through the PPARα receptor (Lo Verme et al. 2004). MAGL hydrolysis of 2-AG also represents an important pathway in the production of free arachidonic acid in the brain that may play a role in neuroinflammation (Nomura et al. 2011). Inhibitors of FAAH and MAGL as well as genetically modified mice that lack these enzymes represent useful tools to elucidate endocannabinoid function, and as a test of proof-of-principle of their potential as therapeutic agents.

## Phytocannabinoids

Cannabis has long been used as a source of fiber for the manufacture of rope and clothing, but this material contains little THC. In contrast, illicit marijuana contains high levels of THC, which has steadily increased from approximately 3 % in the 1980s to 12 % in 2012 (Volkow et al. 2014). In addition to THC, over 100 other cannabinoids have been identified in the particulate phase of the marijuana plant (Husni et al. 2014) and its genome has recently been described (van Bakel et al. 2011). Other cannabinoids of interest including CBD, cannabinol (CBN), and cannabigerol (CBG) largely lack the ability to activate cannabinoid receptors, but are biologically active (Russo 2011). CBD has gained particular interest recently as a constituent in the medication Sativex, which has been found to alleviate spasticity associated with MS (Serpell et al. 2013; Syed et al. 2014), cancer pain in opioid-treated patients (Johnson et al. 2010), and marijuana withdrawal (Allsop et al. 2014). Additionally, CBD is under investigation in assorted clinical trials as an anti-epileptic (Devinsky et al. 2014). Preclinical studies reported that CBD elicits anticonvulsant (Consroe et al. 1982; Martin et al. 1987), anti-inflammatory (Li et al. 2013; Malfait et al. 2000), and anti-tumorgenic (McAllister et al. 2007) effects. Although its mechanism of action remains to be elucidated, it is known to inhibit adenosine uptake (Liou et al. 2008), down-regulate the enzymes FAAH and 5-lipoxygenase (Capasso et al. 2008; Massi et al. 2008), and bind both transient receptor potential vanilloid 1 (TRPV1) (Iannotti et al. 2014) and 5-hydroxytryptamine (serotonin) receptor 1A (5-HT1A) receptors (Russo et al. 2005).

Upon heating, phytocannabinoids rapidly decarboxylate and at the temperature of pyrolysis (200^o^ – 400 ° C) undergo aromatization (Nahas 1993). Polycyclic aromatic hydrocarbons have been identified in marijuana smoke and include higher molecular weight compounds, such as the carcinogens benzo(α)pyrene and benz(α)anthracene. The gas phase of marijuana smoke includes toxic substances including carbon monoxide, hydrogen cyanide, and nitrosamines, which also are present in equivalent concentrations in tobacco smoke (Nahas 1993). THC and other phytocannabinoids are lipophilic and sequester in liver, lung, spleen, and neutral fat (Nahas 1993). THC has an approximate half-life of 8 days in fat and may take up to one month for complete elimination of a single dose in humans (Nahas 1993). Furthermore, THC is a polar compound and is metabolized slowly into more water-soluble, nonpsychoactive metabolites. The bioavailability of inhaled and ingested THC is 20 and 6 %, respectively (Nahas 1993).

## Synthetic Cannabinoids

The purification and structural characterization of THC (Gaoni and Mechoulam 1964) have led to the chemical synthesis of various cannabinoid analogs that have been used extensively in structure–activity relationship studies to characterize cannabinoid-mediated bioactivity (e.g., Johnson and Melvin 1986; Mechoulam et al. 1987), and these efforts contributed directly to the discovery of the cannabinoid receptors (Devane et al. 1988; Herkenham et al. 1990; Matsuda et al. 1990; Munro et al. 1993). The extensive use of synthetic cannabinoid ligands has increased our understanding of the functional relevance and mechanism of action by which phytocannabinoids exert their effects on the immune system. For example, THC has been reported to suppress the antibody response of humans and animals (Klein et al. 1998) and to suppress a variety of activities of T lymphocytes (Kaminski 1998; Klein et al. 2004). Administration of THC to mice also has been reported to inhibit natural killer (NK) cytolytic activity and to reduce interferon gamma (IFNγ) levels (Massi et al. 2000). In addition, THC has been reported to abolish the functional activities of macrophages and macrophage-like cells, including macrophage-mediated cell contact-dependent cytolysis of tumor cells and the processing of antigens (Burnette-Curley et al. 1993; Klein et al. 1991; McCoy et al. 1999). It has been reported also that THC alters the production of chemokines and cytokines, leading to a perturbation in the homeostatic balance between pro-inflammatory (Th_1_) cytokines, which promote systemic inflammation, and anti-inflammatory (Th_2_) cytokines such as IL-4 and IL-10 that play an immunoregulatory role in controlling the inflammatory response. Such a shift in the cytokine profile to a Th_2_ bias may contribute to altered inflammatory responses to infection with bacteria and viruses (Kidd 2003). It is now well recognized that other phytocannabinoids such as CBD and CBN can also alter the functional activities of the immune system (Cabral et al. 2014).

## Immunomodulatory Activity of Cannabinoids

It has been suggested that the CB_1_ has potential to serve as a molecular target for therapeutic attenuation of cognitive impairment and degeneration in select CNS disorders (Pryce et al. 2003; Pryce and Baker 2007; Shen and Thayer 1998), a caveat to this consideration is that activation of this receptor also engenders psychotropic effects, dependence, and cognitive impairment (Jones et al. 1976; Budney et al. 2007; Leweke and Koethe 2008; Vandrey and Haney 2009; Skosnik et al. 2012; Radhakrishnan et al. 2014). However, many neuropathogenic processes are characterized by progressive decline in cognitive functions that are accompanied by, if not caused by, inflammation. Much attention has been focused on CB_2_ not only because of its expression primarily in cells and tissues of the immune system (Munro et al. 1993), but also because of its intricate involvement in immune function and its activation in the whole animal is largely devoid of psychotropic effects (Malan et al. 2003; Kinsey et al. 2011). The level of CB_2_ expression varies among different immune cell populations, with B lymphocytes expressing the highest levels followed by macrophages, monocytes, NK cells, and polymorphonuclear cells, in that order (Galiegue et al. 1995; Schatz et al. 1997). Early studies concluded that the distribution of CB_2_ was confined to peripheral non-neuronal sites. However, it is now recognized that this receptor is expressed by a variety of subsets of immunocompetent cells found in the CNS (Cabral and Marciano-Cabral 2005; Carlisle et al. 2002; Carrier et al. 2004; Fernandez-Ruiz et al. 2007; Nunez et al. 2004; Ramirez et al. 2005). Moreover, the CB_2_ has been reported to be present also on neurons (Van Sickle et al. 2005; Zhang et al. 2014). In general, most of the immunomodulatory effects attributed to THC have been linked to activation of CB_2_.

In this issue, Eisenstein reviews the literature which addresses the effects of cannabinoids on immune function, with an emphasis on T-lymphocytes (Eisenstein 2015). Consistent with the identification of high levels of CB_2_ on cells of the immune system, it is indicated that most of the modulatory effects of THC have been linked functionally to this receptor. Accordingly, it is proposed that selective CB_2_ agonists possess promise as therapeutic agents for treatment of autoimmune diseases and for ablation of graft rejection under conditions of decreased incidence of side effects. The potential of select CB_2_ agonists to ablate graft rejection is particularly relevant to the report by Robinson et al. that explores the mechanism by which agonists selective for CB_2_, such as O-1966, inhibit the Mixed Lymphocyte Reaction (MLR) (Robinson et al. 2015), an in vitro paradigm used as a correlate of organ graft rejection that is mediated predominantly through effects on T-lymphocytes. These investigators observed an increase in the percentage of regulatory T-cells (Tregs) in MLR cultures using mouse spleen cells. Furthermore, pretreatment with an antibody to the anti-inflammatory cytokine IL-10 (anti-IL-10) resulted in a partial reversal of the inhibition of proliferation and blocked the increase of Tregs. Their results bolster the argument that CB_2_-selective agonists may represent useful therapeutic agents to prolong graft survival in transplant patients.

The expression of CB_2_ appears to be modulated in monocytes and macrophages in response to various stimuli (Carlisle et al. 2002). CB_2_ may be particularly responsive to cognate agonists when in a responsive state, i.e., a functional state that is associated with immune cell migratory activity (Carlisle et al. 2002). More work on this issue is clearly needed since the immunomodulatory activity of CB_2_ may be dependent on the activation state of both the target cell population, as well as the vascular endothelial cells at the site of inflammation. This is particularly relevant to the report by Persidsky et al. in which brain microvascular endothelial cells (BMVEC) and monocyte-derived macrophages from human tissue were employed in an in vitro paradigm to show that CB_2_ agonism may represent a strategy for treatment of CNS diseases associated with neuroinflammatory responses (Persidsky et al. 2015). These investigators found that activation of CB_2_ blocks monocyte migration across BMVEC monolayers, dampens LPS-induced secretion of the pro-inflammatory cytokine tumor necrosis-alpha (TNF-α), reduces the expression of a large panel of pro-inflammatory genes activated by TNF-α in BMVEC, and blunts LPS-induced upregulation of genes associated with inflammation in primary human macrophages. Roth and colleagues macrophages report that THC inhibits the differentiation of human monocytes into antigen-presenting dendritic cells (Roth et al. 2015). These investigators report that THC and CB_2_ agonists exert a robust CB_2_-mediated effect on dendritic cells that results in failure to stimulate T cell proliferation or promote maturation into functional effector/memory T cells.

While a large body of data from in vitro studies and animal models indicates that the immunomodulatory activity of THC and CB_2_ agonists can lead to decreased resistance to infectious agents, a comparable linkage in humans has yet to be demonstrated. A major challenge in resolving this issue is that individuals who use marijuana often also use other substances that have immune-suppressing potential. In addition, individuals who use marijuana, or cannabinoid formulations, for therapeutic purposes already possess underlying health conditions that may render them immunocompromised and susceptible to infection. Furthermore, the presence of CBD and other phytocannabinoids in marijuana may counteract the effects of THC and temper the overall immune functional outcome in vitro and in experimental animals. Thus, in order to more closely approximate the human condition, in particular to the impact of cannabinoid exposure on infection, investigators have resorted to the use of primate models. Molina et al. provide a comprehensive review of the consequences of chronic THC or ethanol exposure in rhesus macaques infected with simian immunodeficiency virus (SIV) (Molina et al. 2015) as a model of human immunodeficiency virus (HIV) infection. In comparison to chronic ethanol exposure that produced a plethora of deleterious effects and accelerated progression of end-stage disease, chronic THC exposure resulted in reduced viral load, viral replication, and inflammation. Furthermore, progression to end-stage disease was decreased or not affected. These results highlight the difficulty of translationally applying in vitro outcomes related to effects of cannabinoids on immune function to those anticipated in vivo in the context of an infectious process. Finally, Chen et al. present original research showing that THC produces a modest suppression of HIV gp120-induced IFNγ production through a CB_1_/ CB_2_ independent pathway (Chen et al. 2015). Their results indicate that THC can modulate immune responses through non-cannabinoid receptor targets.

## Immunomodulatory Role of Endocannabinoids

It has been suggested that 2-AG is the cognate functionally-relevant endocannabinoid for CB_2_ (Sugiura et al. 2000; Parolaro et al. 2002). AEA also has been linked to modulation of immune function. However, whether this linkage involves activation of a cannabinoid receptor is uncertain. The immunomodulatory activity mediated by endocannabinoids may occur in an autocrine and paracrine fashion, impacting the functionality of immune cells in a localized environment. Furthermore, such mediated action may be short-lived because of the rapid degradation of endocannabinoids in the intracellular environment. It is now apparent that resident immune cells within the CNS harbor a constitutive endocannabinoid system (Suarez et al. 2010). Thus, it appears that the immediate effective action of endocannabinoids on immune function is at localized sites in the periphery and CNS. It is speculated that, in this context, endocannabinoids play an important role in maintaining the overall “fine tuning” of the immune homeostatic balance within the host.

There is also compelling evidence that the endocannabinoids may provide protective activity, particularly in the brain. However, the basis for the neuroprotection mediated by these endogenous cannabinoids is still rather poorly defined. In this regard, Espejo-Porras et al. describe their characterization of the endogenous cannabinoid system in the transactive response (TAR)-DNA binding protein-43 (TDP-43) transgenic mouse model of ALS during the early symptomatic (70–80 days of age) and postsymptomatic (100–110 days of age) stages (Espejo-Porras et al. 2015). TDP-43 transgenic mice exhibit motor coordination deficits that are accompanied by a loss of motor neurons and reactive microgliosis with increased expression of CB_2_ in the spinal cord. Varied small decreases were found on FAAH expression and increased endocannabinoid levels in spinal cord that were sex- and age-dependent. This initial characterization of the TDP-43 model of ALS sets the stage for testing of pharmacological agents targeting CB_2_, FAAH, or other components of the endocannabinoid system. Thus, these studies may provide the basis for developing intervention strategies for treatment of certain neuroinflammatory diseases. One example of this type of development is described by Mann et al. who report the role of the fatty acyl amino acid (FAAA) palmitoyl-serine (PalmS) in the mouse closed head injury (CHI) model of traumatic brain injury (Mann et al. 2015). PalmS treatment improved neurobehavioral outcome, as indicated by neurological severity score (NSS), which examined reflexes, alertness, coordination, motor abilities and balancing. However, PalmS treatment had no effect on cognitive measures, rotarod performance or levels of biomarkers of brain injury (e.g., lesion volume, edema, or proinflammatory markers). Although PalmS does not bind cannabinoid receptors, its beneficial effects required the presence of CB_2_. These investigators concluded that the reduction in NSS caused by PalmS is mediated by indirect activation of CB_2_ and propose a model that involves receptor palmitoylation, which may result in the structural stabilization of the CB_2_ and enhance its activity.

Nevertheless, at this point the physiological activities of the endogenous cannabinoids remain inadequately defined, and a greater understanding of the functional activities of these agents will be necessary in order to fully develop effective therapeutics. In this regard, Gómez et al. describe the effects of 2-AG on early stage oligodendrocyte progenitor cell (OPC) differentiation (Gomez et al. 2015). These investigators report that basal levels of 2-AG are required to maintain proliferation of early OPCs in vitro. Inhibition of 2-AG degradation with a MAGL inhibitor or exogenous administration of 2-AG, as well as that elicited by selective CB_1_ or CB_2_ agonists, further stimulated early OPC differentiation. The investigators propose a novel mechanism of action for 2-AG in oligodendrocytes coupled to Akt/mTOR signaling, an intracellular pathway important in regulating cell cycle. This exciting work has potential implications in the emerging field of brain repair. Similarly, recent research by Nass et al. shows that MAGL plays a protective role on thermoregulation in mice following endotoxin or cold ambient temperature challenge (Nass et al. 2015). While MAGL inhibition alone had no effect on body temperature in mice, it exerted a profound reduction in core temperature in mice challenged with LPS or cold ambient temperature. In view of these findings, the authors hypothesize that MAGL functions as a protective “brake” from immunological challenges by curtailing 2-AG activation of CB_1_.

## Cannabinoids and Development

The high incidence of cannabis use by adolescents and young adults of childbearing age, raises the question of the potential impact of cannabinoids on brain development. Chronic and/or recurrent use of cannabis may alter brain and/or immune system development and exert effects that are less apparent in adults. A review by Zumbrun et al. in this issue investigates the implications of marijuana use during pregnancy on the offspring (Zumbrun et al. 2015). These authors also address whether maternal or paternal cannabinoid exposure can trigger epigenetic changes, such as altered microRNA, DNA methylation and histone modification, that have long-term immunological implications on offspring and can be carried across generations. Since much of the current data have been derivative of in vivo rodent and in vitro studies, in this review a case is made for the importance of conducting translational research to provide insights applicable to humans. However, it is clear that additional work in this topical area will be necessary in order to develop an understanding of the capacity of cannabis use to alter either neuronal or immune cell development. A review by Moretti et al. describes results of preclinical studies in which the long-term consequences of THC exposure on adolescent mice were examined (Moretti et al. 2015). Whereas THC did not affect levels of brain cytokines in adult mice, it was found to decrease those of proinflammatory cytokines in the adolescent brain. Following a 1.5 month hiatus from the final THC exposure, brain levels of the anti-inflammatory cytokine IL-10 were decreased. These studies demonstrated that chronic exposure of adolescent mice to THC suppressed immunity immediately after treatment. However, after a washout period, THC induced a long-lasting opposite modulation towards a proinflammatory and T-helper-1 phenotype in adulthood, an outcome comparable to that reported previously to occur at peripheral sites. These findings raise the intriguing possibility that cannabis exposure in adolescents leads to increased vulnerability to immune and neuroinflammatory diseases in adulthood. Finally, the report by Cloak et al. in this issue compares salivary cortisol levels, immunological responses (i.e., salivary IL-1β, TNF-α and IL-6 levels), and psychiatric symptoms in adolescent marijuana users and nonusers (Cloak et al. 2015). While cortisol and salivary cytokine levels did not differ between the marijuana users and controls, self-reported and clinician-rated psychiatric (particularly anxiety-related) symptoms were increased in the marijuana users compared with controls. The age of onset was negatively correlated with symptoms and the quantity of lifetime marijuana use was correlated positively with symptoms, while days of abstinence were correlated negatively with symptoms. Although levels of cortisol and cytokines did not correlate with cannabis use or psychiatric symptoms, this work suggests that marijuana use in adolescents may contribute to an aberrant relationship between stress response and psychiatric symptoms.

## Clinical Implications

Cannabinoid agonists exert a variety of effects in the brain on neuronal function and can modulate neuroinflammatory disease levels in several neurodegenerative disease states. Because CB_2_ activation dampens inflammatory responses in the absence of psychotropic effects, it has the potential to serve as a molecular target for attenuating inflammation linked to pathogenic disorders such as MS (Maresz et al. 2007; Zhang et al. 2009b), ischemic/perfusion injury following an induced stroke (Zhang et al. 2007, 2009a), rheumatoid arthritis (Sumariwalla et al. 2004), inflammatory bowel disease (Storr et al. 2008, 2009), inflammatory autoimmune diabetes (Li et al. 2001), spinal cord injury (Adhikary et al. 2011; Baty et al. 2008), sepsis (Tschӧp et al. 2009), autoimmune uveoretinitis (Xu et al. 2007), osteoporosis (Ofek et al. 2006), pain (Kinsey et al. 2011; Malan et al. 2001, 2003; Quartilho et al. 2003; Anand et al. 2009; Deng et al. 2015), hepatic ischemia-reperfusion (I/R) injury (Batkai et al. 2007), and systemic sclerosis (Servettaz et al. 2010). Thus, it is not entirely surprising that cannabinoids alter the antitumor immune response, and McAllister et al. review literature describing the well established antitumor activity of CBD, a cannabinoid that does not bind CB_1_ or CB_2_, in a variety of cancer cell lines, including those derived from glioblastoma, breast, lung, prostate and colon cancer (McAllister et al. 2015). These authors also discuss potential targets of this non-psychoactive phytocannabinoid, as well as the underlying mechanisms of action for its plethora of antitumor effects, elegantly illustrated in Table 1 of their review (McAllister et al. 2015).

Chiurchiu et al. discuss the immunomodulatory effects of cannabinoid signaling on immune cells in the brain (Chiurchiu et al. 2015). The modulatory impact on brain immune responses supports the concept that select cannabinoids have potential as therapeutic agents for management of neuroinflammatory disorders, such as Alzheimer’s disease, MS, Huntington’s disease, amyotrophic lateral sclerosis (ALS), and Parkinson’s disease. The review of Pryce et al. addresses the effects of phytocannabinoids in an experimental autoimmune encephalomyelitis (EAE) model of MS in mice (Pryce et al. 2015). These authors include original data indicating that THC and CBD dampen the behavioral signs and motor deficits associated with EAE. Synthetic CBD was shown to slow down the accumulation of disability from the inflammatory penumbra during relapsing EAE in ABH mice, possibly through blockade of voltage-gated sodium channels. However, while subthreshold doses of each compound given in combination enhanced subjective clinical scores significantly, the experimental conditions applied did not lend themselves to classification of the nature of the drug interaction. In addition, the investigators describe the outcome of a phase III clinical trial aimed at testing the efficacy of oral THC in progressive MS. Although the study did not yield a positive outcome, an a priori analysis of a subgroup of patients who presented with decreased disability revealed that THC reduced disease progression. In addition, CB_2_ agonists have recently been demonstrated to be protective against the reinforcing effects of cocaine in mice (Xi et al. 2011; Zhang et al. 2014). Although activation of CB_2_ produces well described antinociceptive effects in preclinical studies (Ibrahim et al. 2006), the general outcome of clinical trials has been disappointing (Atwood et al. 2012; Dhopeshwarkar and Mackie 2014).

Finally, given the continued controversy surrounding the issue of “medical marijuana”, using the scientific process to discern the safety and efficacy of cannabinoid-based medications remains of paramount importance. To this end, Lynch and Ware provide an in depth analysis of recent clinical trials testing the efficacy of endocannabinoid-based drugs in treating non-cancer pain in humans (Lynch and Ware 2015). A variety of cannabinoids was examined in these studies, including the FDA-approved synthetic cannabinoid receptor agonist nabilone, an oral mucosal cannabis spray, the FAAH inhibitor PF-04457845, oral or inhaled cannabis extract, and smoked cannabis. The majority of these studies revealed modest analgesic effects of these formulations without serious side effects, lending credence to the idea that cannabinoid-based medications ultimately may be a reasonable treatment option for chronic non-cancer pain. However, on a cautionary note, these studies generally have been conducted under conditions of short duration, using relatively small sample sizes and modest effect sizes.

## Conclusions

The potential deleterious effects as well as beneficial uses of cannabis, its phytocannabinoid composition, and synthetic cannabinoid analogs are discussed in these papers. In addition, the role of the endocannabinoid system and approaches to its manipulation to moderate select human disease processes are addressed. It is our aim to place the potential benefits and risks of marijuana use in perspective. As the Guest Editors, we believe this is an excellent opportunity to present the latest works related to this important topic.

## References

[CR1] Adhikary S, Li H, Heller J, Skarica M, Zhang M, Ganea D (2011). Modulation of inflammatory responses by a cannabinoid-2-selective agonist after spinal cord injury. J Neurotrauma.

[CR2] Allsop DJ, Copeland J, Lintzeris N, Dunlop AJ, Montebello M, Sadler C (2014). Nabiximols as an agonist replacement therapy during cannabis withdrawal: a randomized clinical trial. JAMA Psychiatr.

[CR3] Anand P, Whiteside G, Fowler CJ, Hohman AG (2009). Targeting CB2 receptors and the endocannabinoid system for the treatement of pain. Brain Res Rev.

[CR4] Atwood BK, Straiker A, Mackie K (2012). CB2: therapeutic target-in-waiting. Prog Neuro-Psychopharmacol Biol Psychiatry.

[CR5] Baker D, Pryce G, Davies WL, Hiley CR (2006). In silico patent searching reveals a new cannabinoid receptor. Trends Pharmacol Sci.

[CR6] Batkai S, Osei-Hyiaman D, Pan H, El-Assal O, Rajesh M, Mukhopadhyay P (2007). Cannabinoid-2 receptor mediates protection against hepatic ischemia/reperfusion injury. FASEB J.

[CR7] Baty DE, Zhang M, Li H, Erb CJ, Adler MW, Ganea D (2008). Cannabinoid CB2 receptor activation attenuates motor and autonomic function deficits in a mouse model of spinal cord injury. Clin Neurosurg.

[CR8] Blankman JL, Cravatt BF (2013). Chemical probes of endocannabinoid metabolism. Pharmacol Rev.

[CR9] Blankman JL, Simon GM, Cravatt BF (2007). A comprehensive profile of brain enzymes that hydrolyze the endocannabinoid 2-arachidonoylglycerol. Chem Biol.

[CR10] Breivogel CS, Griffin G, Di Marzo V, Martin BR (2001). Evidence for a new G protein-coupled cannabinoid receptor in mouse brain. Mol Pharmacol.

[CR11] Budney AJ, Roffman R, Stephens RS, Walker D (2007). Marijuana dependence and its treatment. Addict Sci Clin Pract.

[CR12] Burnette-Curley D, Marciano-Cabral F, Fischer-Stenger K, Cabral GA (1993). Delta-9 tetrahydrocannabinol inhibits cell contact-dependent cytotoxicity of Bacillus Calmette-Guerin-activated macrophages. Int J Immunopharmacol.

[CR13] Cabral GA, Marciano-Cabral F (2005). Cannabinoid receptors in microglia of the central nervous system: immune functional relevance. J Leukoc Biol.

[CR14] Cabral GA, Raborn ES, Ferreira GA (2014) Phytocannabinoids and the immune system. In: Pertwee R (ed). Handbook of Cannabis. Oxford University Press pp 261–279

[CR15] Capasso R, Borrelli F, Aviello G, Romano B, Scalisi C, Capasso F (2008). Cannabidiol, extracted from Cannabis sativa, selectively inhibits inflammatory hypermotility in mice. Br J Pharmacol.

[CR16] Carlisle SJ, Marciano-Cabral F, Staab A, Ludwick C, Cabral GA (2002). Differential expression of the CB2 cannabinoid receptor by rodent macrophages and macrophage-like cells in relation to cell activation. Int Immunopharmacol.

[CR17] Carrier EJ, Kearn CS, Barkmeier AJ, Breese NM, Yang W, Nithipatikom K (2004). Cultured rat microglial cells synthesize the endocannabinoid 2-arachidonylglycerol, which increases proliferation via a CB2 receptor-dependent mechanism. Mol Pharmacol.

[CR18] Chen W, Crawford RB, Kaplan BLF, Kaminski NE (2015) Modulation of HIVgp120 antigen-specific immune responses in vivo by Δ9-tetrahydrocannabinol. J Neuroimmune Pharmacol. doi:10.1007/s11481-015-9597-x 10.1007/s11481-015-9597-x 25900076

[CR19] Chiurchiu V, Leuti A, Maccarrone M (2015) Cannabinoid signaling and neuroinflammatory diseases: a melting pot for the regulation of brain immune responses. J Neuroimmune Pharmacol. doi:10.1007/s11481-015-9584-2 10.1007/s11481-015-9584-2 25601726

[CR20] Cloak C, Alicata D, Ernst T, Chang L (2015) Psychiatric symptoms, salivary cortisol and cytokine levels in young marijuana users. J Neuroimmune Pharmacol. doi:10.1007/s11481-015-9606-0 10.1007/s11481-015-9606-0 PMC4470746 25875137

[CR21] Consroe P, Benedito MA, Leite JR, Carlini EA, Mechoulam R (1982). Effects of cannabidiol on behavioral seizures caused by convulsant drugs or current in mice. Eur J Pharmacol.

[CR22] Cravatt BF, Demarest K, Patricelli MP, Bracey MH, Giang DK, Martin BR (2001). Supersensitivity to anandamide and enhanced endogenous cannabinoid signaling in mice lacking fatty acid amide hydrolase. Proc Natl Acad Sci U S A.

[CR23] Cravatt BF, Giang DK, Mayfield SP, Boger DL, Lerner RA, Gilula NB (1996). Molecular characterization of an enzyme that degrades neuromodulatory fatty-acid amides. Nature.

[CR24] Deng L, Guindon J, Cornett BL, Makriyannis A, Mackie K, Hohmann AG (2015). Chronic cannabinoid receptor 2 activation reverses Paclitaxel neuropathy without tolerance or cannabinoid receptor 1-dependent withdrawal. Biol Psychiatry.

[CR25] Devane WA, Dysarz FA, Johnson MR, Melvin LS, Howlett AC (1988). Determination and characterization of a cannabinoid receptor in rat brain. Mol Pharmacol.

[CR26] Devane WA, Hanus L, Breuer A, Pertwee RG, Stevenson LA, Griffin G (1992). Isolation and structure of a brain constituent that binds to the cannabinoid receptor. Science.

[CR27] Devinsky O, Cilio MR, Cross H, Fernandez-Ruiz J, French J, Hill C (2014). Cannabidiol: pharmacology and potential therapeutic role in epilepsy and other neuropsychiatric disorders. Epilepsia.

[CR28] Dhopeshwarkar A, Mackie K (2014). CB2 Cannabinoid receptors as a therapeutic target-what does the future hold?. Mol Pharmacol.

[CR29] Di Marzo V, Breivogel CS, Tao Q, Bridgen DT, Razdan RK, Zimmer AM (2000). Levels, metabolism, and pharmacological activity of anandamide in CB(1) cannabinoid receptor knockout mice: evidence for non-CB(1), non-CB(2) receptor-mediated actions of anandamide in mouse brain. J Neurochem.

[CR30] Dinh TP, Carpenter D, Leslie FM, Freund TF, Katona I, Sensi SL (2002). Brain monoglyceride lipase participating in endocannabinoid inactivation. Proc Natl Acad Sci U S A.

[CR31] Dinh TP, Freund TF, Piomelli D (2002). A role for monoglyceride lipase in 2-arachidonoylglycerol inactivation. Chem Phys Lipids.

[CR32] Eisenstein TK (2015) Effects of cannabinoids on T-cell function and resistance to infection. J Neuroimmune Pharmacol. doi:10.1007/s11481-015-9603-3 10.1007/s11481-015-9603-3 PMC4470840 25876735

[CR33] Espejo-Porras F, Piscitelli F, Verde R, Ramos JA, Di Marzo V, de Lago E et al. (2015) Changes in the endocannabinoid signaling system in CNS structures of TDP-43 transgenic mice: relevance for a neuroprotective therapy in TDP-43-related disorders. J Neuroimmune Pharmacol. doi:10.1007/s11481-015-9602-4 10.1007/s11481-015-9602-4 25819934

[CR34] Fernandez-Ruiz J, Romero J, Velasco G, Tolon RM, Ramos JA, Guzman M (2007). Cannabinoid CB2 receptor: a new target for controlling neural cell survival?. Trends Pharmacol Sci.

[CR35] Galiegue S, Mary S, Marchand J, Dussossoy D, Carriere D, Carayon P (1995). Expression of central and peripheral cannabinoid receptors in human immune tissues and leukocyte subpopulations. Eur J Biochem.

[CR36] Gaoni Y, Mechoulam R (1964). Isolation, structure, and partial synthesis of an active constituent of hashish. J Am Chem Soc.

[CR37] Gerard CM, Mollereau C, Vassart G, Parmentier M (1991). Molecular cloning of a human cannabinoid receptor which is also expressed in testis. Biochem J.

[CR38] Gomez O, Sanchez-Rodriguez MA, Ortega-Gutierrez S, Vazquez-Villa H, Guaza C, Molina-Holgado F et al. (2015) A basal tone of 2-arachidonoylglycerol contributes to early oligodendrocyte progenitor proliferation by activating phosphatidylinositol 3-kinase (PI3K)/AKT and the mammalian target of rapamycin (MTOR)pathways. J Neuroimmune Pharmacol. doi:10.1007/s11481-015-9609-x 10.1007/s11481-015-9609-x 25900077

[CR39] Herkenham M, Lynn AB, Johnson MR, Melvin LS, de Costa BR, Rice KC (1991). Characterization and localization of cannabinoid receptors in rat brain: a quantitative in vitro autoradiographic study. J Neurosci.

[CR40] Herkenham M, Lynn AB, Little MD, Johnson MR, Melvin LS, de Costa BR, Rice KC (1990). Cannabinoid receptor localization in brain. Proc Natl Acad Sci U S A.

[CR41] Hermanson DJ, Gamble-George JC, Marnett LJ, Patel S (2014). Substrate-selective COX-2 inhibition as a novel strategy for therapeutic endocannabinoid augmentation. Trends Pharmacol Sci.

[CR42] Howlett AC (2002). The cannabinoid receptors. Prostaglandins Other Lipid Mediat.

[CR43] Howlett AC, Reggio PH, Childers SR, Hampson RE, Ulloa NM, Deutsch DG (2011). Endocannabinoid tone versus constitutive activity of cannabinoid receptors. Br J Pharmacol.

[CR44] Husni AS, McCurdy CR, Radwan MM, Ahmed SA, Slade D, Ross SA (2014). Evaluation of phytocannabinoids from high potency using bioassays to determine structure-activity relationships for cannabinoid receptor 1 and cannabinoid receptor 2. Med Chem Res.

[CR45] Iannotti FA, Hill CL, Leo A, Alhusaini A, Soubrane C, Mazzarella E (2014). Nonpsychotropic plant cannabinoids, cannabidivarin (CBDV) and cannabidiol (CBD), activate and desensitize transient receptor potential vanilloid 1 (TRPV1) channels in vitro: potential for the treatment of neuronal hyperexcitability. ACS Chem Neurosci.

[CR46] Ibrahim MM, Rude ML, Stagg NJ, Mata HP, Lai J, Vanderah TW et al. (2006) CB2 cannabinoid receptor mediation of antinociception. 122(1–2):36–42 10.1016/j.pain.2005.12.018 16563625

[CR47] Jarai Z, Wagner JA, Varga K, Lake KD, Compton DR, Martin BR (1999). Cannabinoid-induced mesenteric vasodilation through an endothelial site distinct from CB1 or CB2 receptors. Proc Natl Acad Sci U S A.

[CR48] Johnson JR, Burnell-Nugent M, Lossignol D, Ganae-Motan D, Potts R, Fallon MT (2010). Multicenter, double-blind, randomized, placebo-controlled, parallel-group study of the efficacy, safety, and tolerability of THC:CBD extract and THC extract in patients with intractable cancer-related pain. J Pain Symptom Manag.

[CR49] Johnson MR, Melvin LS, Mechoulam R (1986). The discovery of nonclassical cannabinoid analgetics. Cannabinoids as therapeutic agents.

[CR50] Jones RT, Benowitz N, Bachman J (1976). Clinical studies of cannabis tolerance and dependence. Ann N Y Acad Sci.

[CR51] Kaminski NE (1998). Regulation of the cAMP cascade, gene expression and immune function by cannabinoid receptors. J Neuroimmunol.

[CR52] Kidd P (2003). Th1/Th2 balance: the hypotesis, its limitations, and implications for health and disease. Altern Med Rev.

[CR53] Kinsey SG, Mahadevan A, Zhao B, Sun H, Naidu PS, Razdan RK (2011). The CB2 cannabinoid receptor-selective agonist O-3223 reduces pain and inflammation without apparent cannabinoid behavioral effects. Neuropharmacology.

[CR54] Klein TW, Friedman H, Specter S (1998). Marijuana, immunity and infection. J Neuroimmunol.

[CR55] Klein TW, Kawakami Y, Newton C, Friedman H (1991). Marijuana components suppress induction and cytolytic function of murine cytotoxic T cells in vitro and in vivo. J Toxicol Environl Health.

[CR56] Klein TW, Newton C, Larsen K, Chou J, Perkins I, Lu L (2004). Cannabinoid receptors and T helper cells. J Neuroimmunol.

[CR57] Lauckner JE, Jensen JB, Chen HY, Lu HC, Hille B, Mackie K (2008). GPR55 is a cannabinoid receptor that increases intracellular calcium and inhibits M current. Proc Natl Acad Sci U S A.

[CR58] Leweke FM, Koethe D (2008). Cannabis and psychiatric disorders: it is not only addiction. Addict Biol.

[CR59] Li K, Feng JY, Li YY, Yuece B, Lin XH, Yu LY (2013). Anti-inflammatory role of cannabidiol and O-1602 in cerulein-induced acute pancreatitis in mice. Pancreas.

[CR60] Li X, Kaminski NE, Fischer LJ (2001). Examination of the immunosuppressive effect of delta9-tetrahydrocannabinol in streptozotocin-induced autoimmune diabetes. Int Immunopharmacol.

[CR61] Liou GI, Auchampach JA, Hillard CJ, Zhu G, Yousufzai B, Mian S (2008). Mediation of cannabidiol anti-inflammation in the retina by equilibrative nucleoside transporter and A2A adenosine receptor. Invest Ophthalmol Visl Sci.

[CR62] Lo Verme J, Fu J, Astarita G, La Rana G, Russo R, Calignano A (2004). The nuclear receptor PPAR-{alpha} mediates the antiinflammatory actions of palmitoylethanolamide. Mol Pharmacol.

[CR63] Lynch M, Ware MA (2015) Cannabinoids for the treatment of chronic non-cancer pain: an updated systematic review of randomized controlled trials. J Neuroimmune Pharmacol. doi:10.1007/s11481-015-9600-6 10.1007/s11481-015-9600-6 25796592

[CR64] Malan TP, Ibrahim MM, Deng H, Liu Q, Mata HP, Vanderah T (2001). CB2 cannabinoid receptor-mediated peripheral antinociception. Pain.

[CR65] Malan TP, Ibrahim MM, Lai J, Vanderah TW, Makriyannis A, Porreca F (2003). CB2 cannabinoid receptor agonists: pain relief without psychoactive effects?. Curr Opin Pharmacol.

[CR66] Malfait AM, Gallily R, Sumariwalla PF, Malik AS, Andreakos E, Mechoulam R (2000). The nonpsychoactive cannabis constituent cannabidiol is an oral anti- arthritic therapeutic in murine collagen-induced arthritis. Proc Natl Acad Sci U S A.

[CR67] Mann A, Smoum R, Trembovler V, Alexandrovich A, Breuer A, Mechoulam R et al. (2015) Palmitoyl serine: an endogenous neuroprotective endocannabinoid-like entity after traumatic brain injury. J Neuroimmune Pharmacol. doi:10.1007/s11481-015-9595-z 10.1007/s11481-015-9595-z 25721934

[CR68] Maresz K, Pryce G, Ponomarev ED, Marsicano G, Croxford JL, Shriver LP (2007). Direct suppression of CNS autoimmune inflammation via the cannabinoid receptor CB1 on neurons and CB2 on autoreactive T cells. Nat Med.

[CR69] Martin AR, Consroe P, Kane VV, Shah V, Singh V, Lander N et al. (1987) Structure-anticonvulsant activity relationships of cannabidiol analogs. In: Rapaka RA, Makriyannis A (eds). Structure-activity relationships of the cannabinoids. U.S. Govt. Printing Office: Washington, D.C. Vol NIDA Res. Monogr. Series #79, pp 48–58 3125480

[CR70] Massi P, Fuzio D, Vigano D, Sacerdote P, Parolaro D (2000). Relative involvement of cannabinoid CB(1) and CB(2) receptors in the Delta(9)-tetrahydrocannabinol-induced inhibition of natural killer activity. Eur J Pharmacol.

[CR71] Massi P, Valenti M, Vaccani A, Gasperi V, Perletti G, Marras E (2008). 5-Lipoxygenase and anandamide hydrolase (FAAH) mediate the antitumor activity of cannabidiol, a non-psychoactive cannabinoid. J Neurochem.

[CR72] Matias I, Pochard P, Orlando P, Salzet M, Pestel J, Di MV (2002). Presence and regulation of the endocannabinoid system in human dendritic cells. Eur J Biochem.

[CR73] Matsuda LA, Lolait SJ, Brownstein MJ, Young AC, Bonner TI (1990). Structure of a cannabinoid receptor and functional expression of the cloned cDNA. Nature.

[CR74] McAllister SD, Christian RT, Horowitz MP, Garcia A, Desprez PY (2007). Cannabidiol as a novel inhibitor of Id-1 gene expression in aggressive breast cancer cells. Mol Cancer Ther.

[CR75] McAllister SD, Soroceanu L, Desprez P-Y (2015) The antitumor activity of plant-derived non-psychoactive cannabinoids. J Neuroimmune Pharmacol. doi:10.1007/s11481-015-9608-y 10.1007/s11481-015-9608-y PMC4470774 25916739

[CR76] McCoy KL, Matveyeva M, Carlisle SJ, Cabral GA (1999). Cannabinoid inhibition of the processing of intact lysozyme by macrophages: evidence for CB2 receptor participation. J Pharmacol Exp Ther.

[CR77] Mechoulam R, Ben-Shabat S, Hanus L, Ligumsky M, Kaminski NE, Schatz AR (1995). Identification of an endogenous 2-monoglyceride, present in canine gut, that binds to cannabinoid receptors. Biochem Pharmacol.

[CR78] Mechoulam R, Devane WA, Breuer A, Zahalka J (1991). A random walk through a cannabis field. Pharmacol Biochem Behav.

[CR79] Mechoulam R, Lander N, Srebnik M, Breuer A, Segal M, Feigenbaum JJ (1987). Stereochemical requirements for cannabimimetic activity. NIDA Res Monogr.

[CR80] Molina P, Amedee AM, Winsauer P, Nelson S, Bagby G, Simon L (2015) Behavioral, metabolic, and immune consequences of chronic alcohol or cannabinoids on HIV/AIDs: studies in the non-human primate SIV model. J Neuroimmune Pharmacol. doi:10.1007/s11481-015-9599-8 10.1007/s11481-015-9599-8 PMC4470723 25795088

[CR81] Moretti S, Franchi S, Castelli M, Amodeo G, Somaini L, Panerai AE et al. (2015) Exposure of adolescent mice to THC induces a long lasting modulation of pro and antinflammatory cytokines in hypothalamus and hippocampus similar to that observed for peripheral macrophages. J Neuroimmune Pharmacol. doi:10.1007/s11481-015-9592-2 10.1007/s11481-015-9592-2 25875136

[CR82] Munro S, Thomas KL, Abu-Shaar M (1993). Molecular characterization of a peripheral receptor for cannabinoids. Nature.

[CR83] Nahas G, Nahas G, Latour C (1993). General toxicity of cannabis. Cannabis:physiology, epidemiology, detection.

[CR84] Nass SR, Long JZ, Scholsburg JR, Cravatt BF, Lichtman AH, Kinsey S (2015) Endocannabinoid catabolic enzymes play differential roles in thermal homeostasis in response to environmental or immune challenge. J Neuroimmune Pharmacol. doi:10.1007/s11481-015-9593-1 10.1007/s11481-015-9593-1 PMC4477849 25715681

[CR85] Nomura DK, Morrison BE, Blankman JL, Long JZ, Kinsey SG, Marcondes MC (2011). Endocannabinoid hydrolysis generates brain prostaglandins that promote neuroinflammation. Science.

[CR86] Nunez E, Benito C, Pazos MR, Barbachano A, Fajardo O, Gonzalez S (2004). Cannabinoid CB2 receptors are expressed by perivascular microglial cells in the human brain: an immunohistochemical study. Synapse.

[CR87] Ofek O, Karsak M, Leclerc N, Fogel M, Frenkel B, Wright K (2006). Peripheral cannabinoid receptor, CB2, regulates bone mass. Proc Natl Acad Sci U S A.

[CR88] Parolaro D, Massi P, Rubino T, Monti E (2002). Endocannabinoids in the immune system and cancer. Prostaglandins Leukot Essent Fatty Acids.

[CR89] Persidsky Y, Fan S, Dykstra H, Reichenbach NL, Rom S, Ramirez SH (2015) Activation of cannabinoid type two receptors (CB2) diminish inflammatory responses in macrophages and brain endothelium. J Neuroimmune Pharmacol. doi:10.1007/s11481-015-9591-3 10.1007/s11481-015-9591-3 PMC4795159 25666933

[CR90] Pertwee RG (2007). GPR55: a new member of the cannabinoid clan?. Br J Pharmacol.

[CR91] Pertwee RG (2009). Emerging strategies for exploiting cannabinoid receptor agonists as medicines. Br J Pharmacol.

[CR92] Pertwee RG, Howlett AC, Abood ME, Alexander SP, Di Marzo V, Elphick MR (2010). International union of basic and clinical pharmacology. LXXIX. cannabinoid receptors and their ligands: beyond CB1 and CB2. Pharmacol Rev.

[CR93] Pryce G, Ahmed Z, Hankey DJ, Jackson SJ, Croxford JL, Pocock JM (2003). Cannabinoids inhibit neurodegeneration in models of multiple sclerosis. Brain.

[CR94] Pryce G, Baker D (2007). Control of spasticity in a multiple sclerosis model is mediated by CB1, not CB2, cannabinoid receptors. Br J Pharmacol.

[CR95] Pryce G, Riddall DR, Selwood DL, Giovannoni G, Baker D (2015) Neuroprotection in experimental autoimmune encephalomyelitis and progressive multiple sclerosis by cannabis-based cannabinoids. J Neuroimmune Pharmacol. doi:10.1007/s11481-014-9575-8 10.1007/s11481-014-9575-8 25537576

[CR96] Quartilho A, Mata HP, Ibrahim MM, Vanderah TW, Porreca F, Makriyannis A (2003). Inhibition of inflammatory hyperalgesia by activation of peripheral CB2 cannabinoid receptors. Anesthesiology.

[CR97] Radhakrishnan R, Wilkinson ST, D’Souza DC (2014). Gone to pot - a review of the association between cannabis and psychosis. Front Psychiatr.

[CR98] Rahn EJ, Hohmann AG (2009). Cannabinoids as pharmacotherapies for neuropathic pain: from the bench to the bedside. Neurotherapeutics.

[CR99] Ramirez BG, Blazquez C, del Gomez PT, Guzman M, de Ceballos ML (2005). Prevention of Alzheimer’s disease pathology by cannabinoids: neuroprotection mediated by blockade of microglial activation. J Neurosci.

[CR100] Robinson RH, Meissler JJ, Fan X, Yu D, Adler MW, Eisenstein TK (2015) A CB2-selective cannabinoid suppresses T-cell activities and increases Tregs and IL-10. J Neuroimmne Pharmacol. doi:10.1007/s11481-015-9611-3 10.1007/s11481-015-9611-3 PMC4528965 25980325

[CR101] Roth MD, Castaneda JT, Kiertscher SM (2015) Exposure to Δ9-tetrahydrocannabinol impairs the differentiation of human monocyte-derived dendritic cells and their capacity for T cell activation. J Neuroimmune Pharmacol. doi:10.1007/s11481-015-9587-z 10.1007/s11481-015-9587-z PMC4470806 25614186

[CR102] Russo EB (2007). History of cannabis and its preparations in saga, science, and sobriquet. Chem Biodivers.

[CR103] Russo EB (2011). Taming THC: potential cannabis synergy and phytocannabinoid-terpenoid entourage effects. Br J Pharmacol.

[CR104] Russo EB, Burnett A, Hall B, Parker KK (2005). Agonistic properties of cannabidiol at 5-HT1a receptors. Neurochem Res.

[CR105] Ryberg E, Larsson N, Sjogren S, Hjorth S, Hermansson NO, Leonova J (2007). The orphan receptor GPR55 is a novel cannabinoid receptor. Br J Pharmacol.

[CR106] Sawzdargo M, Nguyen T, Lee DK, Lynch KR, Cheng R, Heng HH (1999). Identification and cloning of three novel human G protein-coupled receptor genes GPR52, GPR53, and GPR55: GPR55 is extensively expressed in human brain. Brain Res Mol Brain Res.

[CR107] Schatz AR, Lee M, Condie RB, Pulaski JT, Kaminski NE (1997). Cannabinoid receptors CB1 and CB2: a characterization of expression and adenylate cyclase modulation within the immune system. Toxicol Appl Pharmacol.

[CR108] Serpell MG, Notcutt W, Collin C (2013). Sativex long-term use: an open-label trial in patients with spasticity due to multiple sclerosis. J Neurol.

[CR109] Servettaz A, Kavian N, Nicco C, Deveaux V, Chereau C, Wang A (2010). Targeting the cannabinoid pathway limits the development of fibrosis and autoimmunity in a mouse model of systemic sclerosis. Am J Pathol.

[CR110] Shen M, Thayer SA (1998). Cannabinoid receptor agonists protect cultured rat hippocampal neurons from excitotoxicity. Mol Pharmacol.

[CR111] Skosnik PD, D’Souza DC, Steinmetz AB, Edwards CR, Vollmer JM, Hetrick WP (2012). The effect of chronic cannabinoids on broadband EEG neural oscillations in humans. Neuropsychopharmacology.

[CR112] Storr MA, Keenan CM, Emmerdinger D, Zhang H, Yuce B, Sibaev A (2008). Targeting endocannabinoid degradation protects against experimental colitis in mice: involvement of CB1 and CB2 receptors. J Mol Med (Berl).

[CR113] Storr MA, Keenan CM, Zhang H, Patel KD, Makriyannis A, Sharkey KA (2009). Activation of the cannabinoid 2 receptor (CB2) protects against experimental colitis. Inflamm Bowel Dis.

[CR114] Suarez J, Romero-Zerbo SY, Rivera P, Bermudez-Silva FJ, Perez J, de Fonseca FR, Fernandez-Llebrez P (2010). Endocannabinoid system in the adult rat circumventricular areas: an immunohistochemical study. J Comp Neurol.

[CR115] Sugiura T, Kondo S, Sukagawa A, Nakane S, Shinoda A, Itoh K (1995). 2-Arachidonoylglycerol: a possible endogenous cannabinoid receptor ligand in brain. Biochem Biophys Res Commun.

[CR116] SugiuraT WK (2000). 2-Arachidonoylglycerol and the cannabinoid receptors. Chem Phys Lipids.

[CR117] Sumariwalla PF, Gallily R, Tchilibon S, Fride E, Mechoulam R, Feldmann M (2004). A novel synthetic, nonpsychoactive cannabinoid acid (HU-320) with antiinflammatory properties in murine collagen-induced arthritis. Arthritis Rheum.

[CR118] Syed YY, McKeage K, Scott LJ (2014). Delta-9-tetrahydrocannabinol/cannabidiol (Sativex(R)): a review of its use in patients with moderate to severe spasticity due to multiple sclerosis. Drugs.

[CR119] Tschӧp J, Kasten KR, Nogueiras R, Goetzman HS, Cave CM, England LG (2009). The cannabinoid receptor 2 is critical for the host response to sepsis. J Immunol.

[CR120] van Bakel H, Stout JM, Cote AG, Tallon CM, Sharpe AG, Hughes TR (2011). The draft genome and transcriptome of Cannabis sativa. Genome Biol.

[CR121] Vandrey R, Haney M (2009). Pharmacotherapy for cannabis dependence: how close are we?. CNS Drugs.

[CR122] Van Sickle MD, Duncan M, Kingsley PJ, Mouihae A, Urbani P, Mackie K (2005). Identification and functional characterization of brainstem cannabinoid CB2 receptors. Science.

[CR123] Volkow ND, Baler RD, Compton WM, Weiss SR (2014). Adverse health effects of marijuana use. N Engl J Med.

[CR124] Wiley JL, Martin BR (2002). Cannabinoid pharmacology: implications for additional cannabinoid receptor subtypes. Chem Phys Lipids.

[CR125] Xi ZX, Peng XQ, Li X, Song R, Zhang HY, Liu QR (2011). Brain cannabinoid CB(2) receptors modulate cocaine’s actions in mice. Nat Neurosci.

[CR126] Xu H, Cheng CL, Chen M, Manivannan A, Cabay L, Pertwee RG (2007). Anti-inflammatory property of the cannabinoid receptor-2-selective agonist JWH-133 in a rodent model of autoimmune uveoretinitis. J Leukoc Biol.

[CR127] Zhang HY, Gao M, Liu QR, Bi GH, Li X, Yang HJ (2014). Cannabinoid CB2 receptors modulate midbrain dopamine neuronal activity and dopamine-related behavior in mice. Proc Natl Acad Sci U S A.

[CR128] Zhang M, Adler MW, Abood ME, Ganea D, Jallo J, Tuma RF (2009). CB2 receptor activation attenuates microcirculatory dysfunction during cerebral ischemic/reperfusion injury. Microvasc Res.

[CR129] Zhang M, Martin BR, Adler MW, Razdan RK, Jallo JI, Tuma RF (2007). Cannabinoid CB(2) receptor activation decreases cerebral infarction in a mouse focal ischemia/reperfusion model. J Cereb Blood Flow Metab.

[CR130] Zhang M, Martin BR, Adler MW, Razdan RJ, Kong W, Ganea D (2009). Modulation of cannabinoid receptor activation as a neuroprotective strategy for EAE and stroke. J Neuroimmune Pharmacol.

[CR131] Zumbrun E, Sido JM, Nagarkatti PS, Nagarkatti M (2015) Epigenetic regulation of immunological alterations following prenatal exposure to marijuana cannabinoids and its long term consequences in offspring. J Neuroimmune Pharmacol. doi:10.1007/s11481-015-9586-0 10.1007/s11481-015-9586-0 PMC4470789 25618446

